# Characteristics of Sexual Health Programs for Migrants, Refugees, and Asylum Seekers: A Scoping Review

**DOI:** 10.3390/ijerph21080961

**Published:** 2024-07-23

**Authors:** Angelina Bolyta Inthavong, Davoud Pourmarzi

**Affiliations:** National Centre for Epidemiology and Population Health, Australian National University, Canberra 2600, Australia; angelina.inthavong@anu.edu.au

**Keywords:** sexual health, migrants, refugees, asylums seekers, education, HIV, sexually transmitted infections

## Abstract

Background: Social, cultural, and structural determinants of health for migrants, refugees, and asylum seekers can contribute towards poorer sexual health outcomes. People from migrant, refugee, and asylum seeker backgrounds usually use less mainstream sexual healthcare services and have lower levels of sexual health literacy compared with the destination countries’ general population. However, there is little collated knowledge about sexual health programs that have been implemented to improve sexual health among these groups. This study aimed to describe the characteristics of programs for improving sexual health among migrants, refugees, and asylum seekers. Methods: This study was a scoping review in which Scopus and PubMed were searched for peer-reviewed articles published in English since 2010 from countries similar to and including Australia such as New Zealand, the United States of America, Canada, the United Kingdom, and European Union countries). The final search of databases was performed on 26 June 2023, and resultant studies were inputted into Endnote and Covidence systematic review software to remove duplicates before screening. The study utilised a qualitative approach using inductive thematic analysis of the content of the studies to extract and categorise the characteristics of the program. Results: A total of 3044 articles were screened and 20 were included for data extraction. The included studies were conducted in six countries (the United States of America, Australia, France, Italy, Denmark, and Belgium). The key characteristics of sexual health programs identified included consumer consultation and engagement, cultural appropriateness, language support, peer education, self-directed learning, group learning, financial support, and outreach services. the programs had a combination of these characteristics to ensure that they can improve target population engagement with educational materials and decrease barriers to accessing sexual healthcare. Conclusion: Consideration of migrants, refugees, and asylum seekers’ culture, language, socioeconomic characteristics, and access to information and services in the design of the sexual health programs may improve sexual health outcomes among these groups. However, further research is needed to assess the importance and implementation feasibility of the identified characteristics for sexual health programs in specific context.

## 1. Introduction 

With the increasing threat of climate change and international tensions, the number of international migrants worldwide is set to increase from the current figure of 281 million [1]. Migrants, refugees, and asylum seekers make up a large percentage of Australia’s population, with 7.5 million (29.1%) of the population born overseas [2]. Australian migrants, refugees, and asylum seekers are essential contributors to society and necessary for growth and development [3]. 

There is no formal definition of an international migrant, but most experts agree that ‘an international migrant is someone who changes his or her country of usual residence, irrespective of the reason for migration or legal status’ [4]. Refugees are generally defined as ‘persons who are outside their country of origin for reasons of feared persecution, conflict, generalized violence, or other circumstances that have seriously disturbed public order and, as a result, require international protection’ and asylum-seekers as ‘individuals who have sought international protection and whose claims for refugee status have not yet been determined’ [5]. Internally displaced people are defined as ‘persons or groups of persons who have been forced or obliged to flee… and who have not crossed an internationally recognized state border’ [6]. 

The experience of migration is a key determinant of health, with migrants, refugees, and asylum seekers possessing poorer physical and mental health outcomes. A key determinant of poorer health for migrants, refugees, and asylum seekers is their experiences of barriers to accessing health services in their destination country [7,8,9]. Depending on their migration or refugee status their ability to access health services, resources and information can differ [10,11]. For instance, due to financial circumstances and eligibility to access services, migrants are often able to afford more medical services in comparison to refugees and asylum seekers. Sexual health is one of the issues that this population may experience barriers to accessing services in their destination country [11,12]. 

Sexual health is a cornerstone to achieving Sustainable Development Goal 3, ‘good health and well-being’ through universal access to healthcare [13]. It is fundamental to the overall health and well-being of individuals, couples, and families and the social and economic development of communities and countries [14,15]. The World Health Organization (WHO) defines good sexual and reproductive health as a state of complete physical, mental, and social well-being in all matters relating to sexuality and the reproductive system [16]. All individuals have a right to bodily autonomy and access to services that support that right [17]. However, many populations face challenges in exercising their autonomy over their sexual health due to their gender, sexuality, race, ethnicity, age and/or diverse physical and mental abilities [18,19]. Good sexual and reproductive health implies that people are able to have pleasurable and safe sexual experiences, the capability to reproduce and the freedom to decide if, when, and how often to do so free of coercion, discrimination, and violence [20]. 

Social, cultural, and structural determinants of health for migrants, refugees, and asylum seekers contribute to poorer sexual and reproductive health outcomes [21,22]. Social determinants range across age, gender, ethnicity, social class, employment, country of origin, destination country and migration status [23], whereas cultural determinants encompass cultural norms, religious norms, language barriers, health traditions, influence of family and community members, fear of discrimination, and stigmatisation by healthcare providers [24]. Structural determinants move beyond individuals, focusing rather on systems, policies, institutions, and regulations that establish legal and administrative barriers, stigma and discrimination, inadequate resourcing, financial barriers, and poorer living and working conditions [25]. These key determinants of health result in limited access to contraception and abortion services and increased risk of unintended pregnancy, sexually transmitted infections, reproductive tract infections, female genital mutilation, and sexual and gender-based violence [12]. People from migrant, refugee and asylum seeker backgrounds usually use less mainstream sexual health services and have lower levels of sexual health literacy compared with the destination countries’ general population [26]. As a result, effective and acceptable sexual health programs are integral in combatting determinants of poor sexual health for migrants, refugees, and asylum seekers. In improving the effectiveness and acceptability of such programs, it is important that characteristics of these populations are considered in the program designs and delivery.

There is little collated knowledge about the characteristics of sexual health programs that have been implemented to improve migrants’, refugees’ and asylum seekers’ sexual health [27]. Therefore, the aim of this scoping review is to describe the characteristics of the design and implementation of programs aimed at improving sexual health among people with migrant, refugee, and asylum seeker backgrounds and to provide recommendations for future program design and delivery in this area.

## 2. Methods

A scoping review methodology was designed based on the JBI methodology for scoping review guidelines [28] to answer the question: ‘What are the characteristics of sexual health programs for migrants, refugees, and asylum seekers?’. The Preferred Reporting Items for Systematic Reviews and Meta-Analyses Extension for Scoping Reviews (PRISMA-ScR) criteria guided the scoping review [29]. The protocol for this review is available as a pre-print (https://doi.org/10.13140/RG.2.2.13342.31045/2, accessed on 18 July 2024).

### 2.1. Inclusion and Exclusion Criteria 

#### 2.1.1. Participants

The scoping review investigated programs that focused on people with migrant, refugee, and asylum seeker backgrounds resettling in another country. The participants included in the search strategy and inclusion category terms for this scoping review were identified based on the definitions approved by the United Nations High Commissioner for Refugees (UNHCR) [4,5]. In this review, programs focused on internally displaced people were not included [6].

#### 2.1.2. Concept

Studies reporting sexual health programs for migrants, refugees, and asylum seekers were used in this scoping review. The definition of sexual health used for the search strategy and inclusion criteria were obtained from the four sexual health program areas outlined in the WHO framework [30]. This review focused on two areas, including comprehensive education and information, support, and care and prevention and control of human immunodeficiency virus (HIV) and other sexually transmitted infections (STIs). The sexual and gender-based violence prevention and sexual function and psychosexual counselling dimensions were not included in the scoping review eligibility criteria due to the limitations of the authors’ specialised areas [30]. 

#### 2.1.3. Context

This review included studies from the destination countries of migrants, refugees, and asylum seekers. This review included studies from countries similar to and including Australia such as New Zealand, the United Kingdom, the United States of America, Canada, and European Union countries.

#### 2.1.4. Types of Sources

Peer-reviewed articles published in English that used any quantitative, qualitative, and mixed-method design were included. In addition, only articles published from 2010 were considered in the scoping review due to the relevancy of interventions and context of migration. However, review articles, grey literature, and opinion papers were not included. 

#### 2.1.5. Search Strategy

An initial search of two databases, PubMed and Scopus, was conducted to identify whether there were articles on the topic. The generated relevant articles were used to design a full search strategy by using text words found in the titles and abstracts, and the index terms used to describe the articles. The search was conducted using the keywords on PubMed and Scopus. A manual search of the reference lists of included papers in the full-text review was conducted to identify additional sources missed from the original search in order to conduct a thorough scoping review. The final search of databases was performed on 26 June 2023.

#### 2.1.6. Study Selection

After the search of databases, published studies were inputted into Endnote and then the Covidence systematic review software to remove duplicates. Titles and abstracts were then screened in Covidence by one reviewer and assessed against inclusion criteria with the guidance of a second reviewer. The full text of relevant titles and abstracts were then assessed against inclusion criteria. Any reasons for excluding full-text papers were recorded and discussed with the second reviewer.

#### 2.1.7. Data Extraction and Analysis

Data were extracted by one reviewer with the assistance of a second author. For each study, data related to the program and setting were extracted. Additional extracted data included year, country, targeted population, sexual health area, type of program, methods of evaluation, and general statements from authors about the outcomes of the program found in the conclusion section of the articles. 

The study utilised a qualitative approach, using inductive thematic analysis of the content of the studies related to the description of the programs. In order to analyse the data, six phases of thematic analysis were used [31]. 

The reviewer was familiarised with the data by first reviewing each study by reading through the Introduction, Methods, Results, and other parts of the study thoroughly. Analysis of the data then took place by isolating the parts of the study detailing the program and rereading to enable deeper thinking and the generation of initial codes. The initial codes were then discussed with the second reviewer. The reviewers then worked in multiple rounds to categorise the codes and develop themes, then review them before defining and naming themes.

## 3. Results 

**Figure 1:**
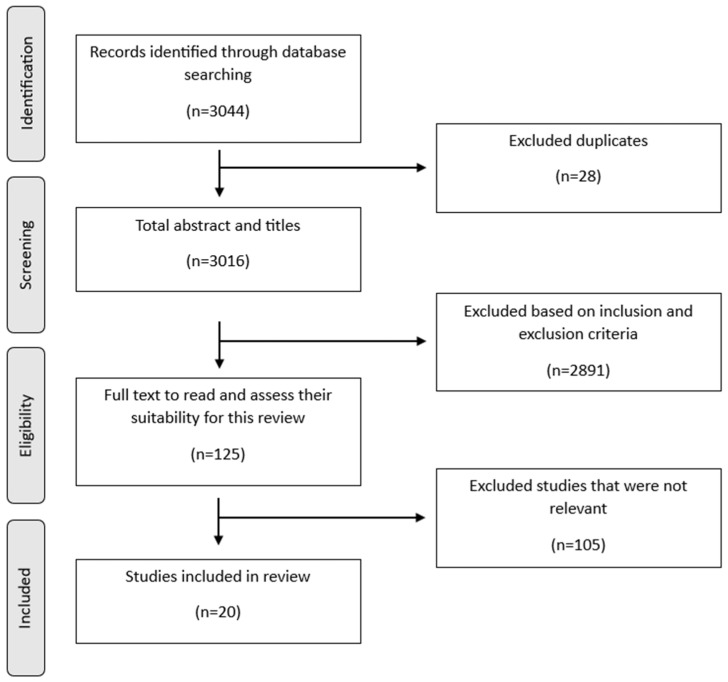
PRISMA diagram of scoping review on programs for improving sexual health among migrants, refugees, and asylum seekers.

The search strategy retrieved a total of 3044 studies. Once duplicates were removed, 3016 studies’ titles and abstracts were screened for eligibility. The full text of 125 studies was screened, of which 105 studies were excluded. This left 20 studies to be included in this review (Figure 1). 

### 3.1. Descriptions of the Included Studies

The 20 included studies were conducted in six countries including the United States of America, Australia, France, Italy, Denmark, and Belgium. The majority of the studies were from the United States of America (*n* = 10) [32,33,34,35,36,37,38,39,40,41] and Australia (*n* = 5) [42,43,44,45]. Out of the two sexual health areas being investigated, the majority of the studies were conducted on STI and/or HIV prevention and control (*n* = 18) [32,33,34,35,36,38,39,40,41,42,43,45,46,47,48,49,50,51]. Only two of the included studies focused on comprehensive education and information, support, and care (*n* = 2) [37,44]. Most of the studies used the term ‘migrants’ without providing details if they also included refugees and asylum seekers (*n* = 17) [32,33,34,35,36,38,39,40,41,44,45,46,47,48,50,51]. Three studies reported programs specifically for refugees (*n* = 3) [37,42,43,49], and one reported on undocumented migrants (*n* = 1) [47] (Table 1).

### 3.2. Characteristics of the Programs

The included studies reported different characteristics of programs to improve engagement and access to sexual health education and services. These characteristics included consumer consultation and engagement, cultural appropriateness, language support, peer education, self-directed learning, group learning, financial support, and outreach services. Most of these characteristics were interconnected and used together in most of the programs. 

### 3.3. Consumer Consultation and Engagement

Seven studies reported using the approach of consumer consultation and engagement in program development [32,33,36,40,41,45,46]. Three studies conducted focus groups with people from the target population [32,36], and a mixture of community members and key community partners [40] to include the knowledge, attitudes, and cultural beliefs of the community to aid in the development of effective and acceptable programs. Another three studies integrated participatory research frameworks into their study design, which often included a combination of researchers, relevant non-governmental organisations, healthcare professionals, and community members [41,45,46], whilst Chen’s study specifically collaborated with two mothers to develop their stories into materials for the program [33].

### 3.4. Cultural Appropriateness 

Cultural appropriateness of the program was determined as an essential characteristic in seven studies due to its capacity to increase the acceptability and usability of programs [36,40,41,42,43,44,46]. Three studies were found to consult community members to ensure that the terminology in the programs was worded in a culturally sensitive manner and/or that language translations were correct [40,42,43]. Cultural values and traditions were integrated into the content of two programs with the aid of community consultation [36,41]. Three programs involved community members in checking that the programs’ technique and recruitment reflected the population of interest’s background, living conditions, and lifestyle [41,44,46].

### 3.5. Language Support

In five studies, some form of language support was provided to aid migrants, refugees, and asylum seekers in engaging with the programs [32,34,36,43,45]. Two studies developed information in the participant’s preferred language [32,43]. Alternatively, three other studies chose to work with bilingual facilitators and educators who were able to provide language support [34,36,45,51]. 

### 3.6. Peer Education 

Eight studies used peer education methods to improve the effectiveness and acceptability of their programs [33,34,36,38,39,40,42,48]. Most of this program involved peer educators who were bilingual or/and bicultural (from the same cultural background), which enabled participants to feel comfortable and to improve their engagement with the educational materials [34,36,38,39,42,48]. In addition, in some programs, these bilingual and bicultural peer educators also possessed previous experiences or qualifications in health and/or education [34,36,42]. Three studies reported the requirement of the peer educators to have additional connections with participants such as similar experiences of motherhood [33], being part of a sport team with participants [38], or having influential positions in the community [42]. The selection of peer educators in four studies possessed a gender focus where the recruitment involved only women, only men, or both men and women to facilitate separate workshops to prevent ‘gender awkwardness’ [34,36,38,42]. 

### 3.7. Self-Directed Learning

A key characteristic identified as important in five studies was self-directed learning methods used for educational programs [32,33,35,41,46]. Self-directed learning took various forms such as the availability of educational materials in DVD format [32] or online on websites that provided for the target groups [41]. Two studies also used story-telling videos that were available online for participants [33,35]. The study argued that self-directed learning was an effective element due to its ability to provide private and self-directed learning at a convenient time and place. 

### 3.8. Group Learning 

Six studies reported group learning as a characteristic of their educational programs [35,37,38,39,42,44]. In three studies, teaching involved sharing stories and role-playing [38,39,44]. In one of these studies, role-playing took place between groups and was elevated with dance [44]. All of these group learnings were face-to-face, with the exception of one that met virtually because of the COVID-19 pandemic [35,37]. 

### 3.9. Financial Support

Nine studies reported the characteristics of financial support that were seen to increase participation [33,34,37,42,43,48,49,50,51]. Four studies compensated participants for their time [33,34,37,42] whether it was through renumerated money or gift cards ranging from $5 USD [34] to $200 AUD [42].

Another large portion of studies found that offering free-of-charge health assessments, testing, and treatment decreased barriers to accessing sexual healthcare [34,43,48,49,50,51]. Five studies provide free health assessments along with STI and HIV screening [34,43,48,49,50,51]. 

### 3.10. Outreach Services

Outreach services including testing and education were reported in four different programs [34,46,47,50]. Two programs involved the model of outreach testing at community locations which were convenient and popular for reaching target population groups [46,50]. Two other programs used the outreach testing model at places of work and stay to engage with specific groups of populations such as undocumented migrants and sex workers [34,47].

## 4. Discussion 

The review identified different sexual health programs that targeted various populations of migrants, refugees and asylum seekers population. These programs used different strategies and included various characteristics to improve their outcomes. The key characteristics included consumer consultation and engagement, cultural appropriateness, language support, peer education, self-directed learning, group learning, financial support, and outreach services. 

The two most popular characteristics included in programs were financial support and peer education, which assisted in overcoming barriers to participation [33] and improved engagement with educational materials [38]. Other vital elements of sexual health programs such as consumer consultation and engagement, cultural appropriateness, and language support all share the goal of increasing the acceptability and usability of programs [41]. These are integral elements for successful sexual health programs for migrants, refugees, and asylum seekers in order to address the social, cultural, and structural determinants of health that contribute towards poorer sexual health outcomes [21].

Characteristics such as consumer consultation and engagement, cultural appropriateness, and language support were interlinked. To include these characteristics, the program designs involved consulting community members to ensure that cultural values and traditions were integrated into the programs [36,41]. Cultural appropriateness and language support also possessed similarities with community consultation, ensuring that language translations were correct and appropriate [40,42,43], and information was developed in the participants’ respective preferred languages in order to improve engagement with the programs [32,43]. Therefore, characteristics such as consumer consultation and engagement as well as language support all involved similar techniques and goals to create a culturally tailored and effective program for migrants, refugees, and asylum seekers. Culturally tailored health programs have been previously identified as integral for the effective participation and engagement of migrants, refugees, and asylum seekers as they are culturally respectful and person-centred, promote healthy lifestyles, increase family and community support, and increase knowledge [52]. This is especially important in the area of sexual health, which is considered a sensitive topic. Consumer consultation and engagement are effective in engaging migrants with similar sensitive topics such as drug misuse, migrants treated as research partners enabled the development of trust and rapport whilst also improving engagement with the program and outcomes by explaining how findings can be used to benefit the community directly [53]. Similar sensitive services such as mental health services have also been found that when health providers lacked cultural and spiritual awareness, they were not able to provide culturally appropriate information decreasing engagement with services [54].

Several studies also combined characteristics such as group learning, peer educators, and language support to deliver programs for migrants, refugees, and asylum seekers. Studies utilised peer educators to conduct group learning. This enabled participants to feel comfortable and to improve their engagement with the educational materials [34,36,38,39,42,48]. Peer educators have long been recognised as an important resource for improving the continuity of care for new refugees and migrants due to their knowledge of the healthcare system and their role in the community [55]. Participants and peer educators are more likely to share similar values and might induce behaviour via ‘identification’ or ‘internalisation’ [56]. Furthermore, peer educators who can provide language support increase the availability of multilingual health materials overcoming language and communication barriers to increase the utilisation of health services and screening among migrants [57].

The characteristic financial support and outreach services were also combined in some studies with free-of-charge testing. Programs that offered services free of charge decreased barriers to accessing sexual healthcare [34,43,48,49,50,51]. Outreach testing similarly aimed to remove barriers to access by going to convenient and popular community locations to reach target population groups [46,50]. Therefore, both financial support and outreach testing elements being present in two studies improved access and engagement with sexual health services by overcoming common barriers [34,43,48,49,50,51]. This is corroborated by previous literature which suggested that challenges such as high costs need to be addressed in order for migrants, refugees, and asylum seekers to be able to access health services due to prioritisation of other needs [58]. Migrants, refugees, and asylum seekers face structural disadvantages and vulnerabilities such as socioeconomic difficulties and as a result may not be able to attend workshops, as they will need to be away from work and not getting paid. However, this experience is heterogeneous, with each group experiencing different financial circumstances [59]. For instance, migrants are often able to afford more medical services in comparison to refugees and asylum seekers. In addition, the six included countries (Australia, the United States of America, France, Italy, Denmark, and Belgium) possess very different medical systems with varying levels of financial burden and eligibility criteria based on type of visa and migration status for accessing sexual health services [60,61]. As a result, free-of-charge outreach testing can remove structural barriers and increase engagement with health programs [34].

## 5. Strengths and Limitations

This scoping review provides a quality and comprehensive summary of the characteristics of sexual health programs for migrants, refugees, and asylum seekers. It focused only on peer-reviewed studies from countries similar to the Australian context such as the United Kingdom, the United States of America, Canada, and European countries so that the findings of this scoping review were specific and applicable to possible policy recommendations. However, since studies not written in English were excluded this limited the availability of information from studies such as European countries possibly meaning results are more relevant for the United States of America and Australia which produced a significant proportion of this scoping review’s literature. In addition, since grey literature was not searched, this limits the scoping review’s findings. Furthermore, as some of the studies did not specify the type of migrants that participated, a weakness of the study is that it is difficult to identify if those findings were also relevant to refugees and asylum seekers. Most papers included were on HIV and HPV prevention and control, with not much on comprehensive sex education, which also limits the scoping review. In this review, we did not assess the quality of the studies as the aim was to extract the elements of the program not the assessment of the outcomes reported. Additionally, we did not report the outcome and relied on the conclusion reported in the articles. 

## 6. Conclusions 

Consumer consultation and engagement, cultural appropriateness, language support, peer education, self-directed learning, group learning, financial support, and outreach services were characteristics of sexual health programs for migrants, refugees, and asylum seekers. Studies used multiple elements together with popular combinations involving consumer consultation and engagement, with cultural appropriateness, and language support to ensure they were culturally tailored. Another common mixture of characteristics integrated into programs was group learning, peer educators and language support to improve target population engagement with the educational materials. Financial support and outreach services elements also decreased barriers to accessing healthcare. The findings of this scoping review have identified common characteristics of sexual health programs that can act as possible policy or program recommendations to improve sexual health outcomes for migrants, refugees, and asylum seekers in countries with similar context to the included studies. The characteristics identified in this review can be implemented in programs to improve their outcomes. Further research is needed to assess the importance and implementation feasibility of the identified characteristics in specific context.

## Figures and Tables

**Table 1:** Descriptions of the included studies in the scoping review on programs for improving sexual health among migrants, refugees, and asylum seekers.

Study	Country	Population	Sexual Health Area	Type of Program	Methods of Evaluation	Conclusions
Dianne Morrison-Beedy et al., 2023 [37]	United States	Refugee adolescent girls 15–17 years old from Asia, Africa, and Middle East	Comprehensive education and information, support, and care	Virtual sexual health promotion program	Interviews	Girls felt comfortable sharing and learning in groups.
Meagan Roberts et al., 2017 [44]	Australia	Southeast Asian, African, and Middle Eastern migrants	Comprehensive education and information, support, and care	Sharing stories, youth theatre program	Pre-evaluation and post-evaluation scenarios	Youth drama workshops improved sexual health knowledge.
Armando Valdez et al., 2015 [32]	United States	Latino and Korean–American parents	STI prevention and control	Culturally and linguistically appropriate HPV vaccine education delivered using DVDs	Pre- and post-test using randomized controlled design	The intervention improved vaccine uptake among migrants.
Peter D Drummond et al., 2011 [42]	Australia	West African refugees	HIV and STI prevention and control	Peer-educator led workshop to increase sexual health knowledge	Pre-intervention and post-intervention questionnaires	Improved discussion surrounding sexual health topics generally considered taboo.
Angela Chia-Chen Chen et al., 2022 [33]	United States	Vietnamese–American mothers	STI prevention and control	Digital storytelling program for improving mothers’ intention to vaccinate their children against HPV.	Pre-intervention and post-intervention questionnaires	Increased HPV vaccinations for children.
Anne Gosselin et al., 2019 [46]	France	Sub-Saharan African or non-French–Caribbean immigrants	HIV and STI prevention and control	Outreach rapid HIV testing and personalised sessions on sexual health using motivational interviewing techniques.	Pre-intervention and post-intervention questionnaires	Improved immigrants’ empowerment in sexual health.
Fredrikke C Knudtzen et al., 2022 [47]	Denmark	Undocumented migrants	STI prevention and control	Community outreach program	Evaluation method not described	Outreach programs were able to reach increased numbers of patients.
Tullio Prestileo et al., 2021 [48]	Italy	New arrival African migrants	HIV and STI prevention and control	Screening program for HBV, HCV, and HIV	Evaluation method not described	Infectious disease screening programs for migrants were effective.
Kathleen Prokopovich et al., 2023 [45]	Australia	Macedonian migrants	STI prevention and control	School-based HPV vaccination	Evaluation method not described	Public trust in schools increased vaccinations for migrant communities.
Arlene C. Seña et al., 2010 [34]	United States	Latino immigrants	HIV and STI prevention and control	Door-to-door rapid HIV testing	Pre-intervention survey only	Door-to-door rapid HIV testing was feasible and accepted by migrants.
Curtis Chan et al., 2022 [43]	Australia	Overseas-born adults at risk of acquiring HIV	HIV and STI prevention and control	Program providing free PrEP	Survey at enrolment	Access to free PrEP and translated information for Medicare-ineligible people created high adherence to PrEP.
Sunny Wonsun Kim et al., 2023 [35]	United States	Vietnamese and Korean–American mothers	STI prevention and control	Digital storytelling workshops for child HPV vaccination	Pre-intervention and post-intervention surveys	The workshop was a feasible and accepted method of increasing HPV vaccination.
Veronica C Hoad & Aesen Thambiran, 2012 [49]	Australia	Refugees	STI prevention and control	Chlamydia and Gonorrhoea screening	Evaluation method not described	Screening was most useful for sexually active refugees up to the age of 39.
Britt Rios-Ellis et al., 2010 [36]	United States	Latino	HIV and STI prevention and control	Culturally and linguistically relevant HIV prevention curriculum delivered at community location sessions	Pre-intervention and post-intervention survey	Increased knowledge of sexual health and intention to practice safer sex.
Jasna Loos et al., 2016 [50]	Belgium	Sub-Saharan African migrants	HIV and STI prevention and control	Community-based outreach HIV-testing	Observations and informal interviews	Outreach HIV testing was feasible and reduced barriers to uptake of testing.
Bahar Azadi et al., 2021 [51]	France	Migrant men	HIV and STI prevention and control	Voluntary HIV and hepatitis testing	Interviews	Most participants were satisfied with screening.
Omar Martinez et al., 2014 [38]	United States	Latino men	HIV and STI prevention and control	Community-level social network: health education, small group didactic sessions and no-cost screening for HIV and STIs	Post-intervention evaluation module	Increased sexual health knowledge among migrants.
Nilda Peragallo Montano et al., 2018 [39]	United States	Hispanic women	HIV and STI prevention and control	Culturally tailored intervention: small groups	Pre-intervention and post-intervention survey	Reduced HIV/STI risk-related behaviour.
Jesús Sánchez et al., 2013 [40]	United States	Hispanic migrant workers	HIV and STI prevention and control	Adapted stage-enhanced motivational interviewing	Pre-intervention and post-intervention surveys	Increased HIV-preventative behaviours.
Hee Yun Lee et al., 2023 [41]	United States	Hmong–Americans	STI prevention and control	Web-based eHealth educational website	Pre-intervention and post-intervention surveys	Improved knowledge of and uptake of HPV vaccinations.

## Data Availability

The datasets generated and/or analysed during the current study are available from the corresponding author on reasonable request. Inthavong A, Pourmarzi D. Describing Sexual Health Interventions for Migrants and Refugees: A Scoping Review Protocol. 2023; Pre-print. https://doi.org/10.13140/RG.2.2.13342.31045/2. Available from: https://www.researchgate.net/publication/378709377_Describing_Sexual_Health_Interventions_for_Migrants_and_Refugees_A_Scoping_Review_Protocol. (accessed on 18 July 2024).
